# Similar NF-κB Gene Signatures in TNF-α Treated Human Endothelial Cells and Breast Tumor Biopsies

**DOI:** 10.1371/journal.pone.0021589

**Published:** 2011-07-06

**Authors:** Martine Perrot-Applanat, Sophie Vacher, Aurore Toullec, Irma Pelaez, Guillaume Velasco, Françoise Cormier, Hanan El Sheikh Saad, Rosette Lidereau, Véronique Baud, Ivan Bièche

**Affiliations:** 1 INSERM U965, Paris, France; 2 Université Paris-Diderot, Sorbonne Paris Cité, Paris, France; 3 INSERM U735, Institut Curie - Hôpital René Huguenin, FNCLCC, Saint-Cloud, France; 4 INSERM U1016, Institut Cochin, Paris, France; 5 CNRS UMR8104, Paris, France; 6 Université Paris Descartes, Paris, France; University of Tor Vergata, Italy

## Abstract

**Background:**

Endothelial dysfunction has been implicated in the pathogenesis of diverse pathologies ranging from vascular and immune diseases to cancer. TNF-α is one of the mediators of endothelial dysfunction through the activation of transcription factors, including NF-κB. While HUVEC (macrovascular cells) have been largely used in the past, here, we documented an NF-κB gene signature in TNFα-stimulated microvascular endothelial cells HMEC often used in tumor angiogenesis studies.

**Methodology/Principal Findings:**

We measured mRNA expression of 55 NF-κB related genes using quantitative RT-PCR in HUVEC and HMEC. Our study identified twenty genes markedly up-regulated in response to TNFα, including adhesion molecules, cytokines, chemokines, and apoptosis regulators, some of them being identified as TNF-α-inducible genes for the first time in endothelial cells (two apoptosis regulators, TNFAIP3 and TNFRSF10B/Trail R2 (DR5), the chemokines GM-CSF/CSF2 and MCF/CSF1, and CD40 and TNF-α itself, as well as NF-κB components (*RELB*, *NFKB1*or *50/p105* and *NFKB2* or *p52/p100*). For eight genes, the fold induction was much higher in HMEC, as compared to HUVEC. Most importantly, our study described for the first time a connection between NF-κB activation and the induction of most, if not all, of these genes in HMEC as evaluated by pharmacological inhibition and RelA expression knock-down by RNA interference.

Moreover, since TNF-α is highly expressed in tumors, we further applied the NF-κB gene signature documented in TNFα-stimulated endothelial cells to human breast tumors. We found a significant positive correlation between *TNF* and the majority (85 %) of the identified endothelial TNF-induced genes in a well-defined series of 96 (48 ERα positive and 48 ERα negative) breast tumors.

**Conclusion/Significance:**

Taken together these data suggest the potential use of this NF-κB gene signature in analyzing the role of TNF-α in the endothelial dysfunction, as well as in breast tumors independently of the presence of ERα.

## Introduction

Tumor necrosis factor (TNF-α) is a potent multi-functional cytokine produced by various cells, including neutrophils, activated macrophages, T and natural killer cells, and fibroblasts, in response to inflammation, infection, tissue injury and other environmental challenges. TNF-α elicits a large number of biological activities including the control of cell proliferation, differentiation and apoptosis, and is an important mediator of inflammation and immunity [1]. Considerable progress has been made in understanding the molecular mechanisms that mediate TNF-α-induced cellular responses. TNF-α binds to membrane receptors, TNFR1 (or TNF-Rp55) and TNFR2 (or TNF-Rp75), which initiate signalling pathway leading to gene induction *via* activation of transcription factors [2]–[3]. TNFR1 is ubiquitously expressed, while TNFR2 expression is restricted to endothelial and hematopoietic cells [4]. Through complex signaling cascades and networks, signal transducers lead to the activation of transcription factors (NF-κB, AP-1 and SP1) [2] and their binding to the promoters of specific genes.

The transcription factor NF-κB is a key inducible transcription factor which regulates numerous genes controlling inflammation, immune response, cell growth, tissue differentiation and apoptosis [5]–[7]. NF-κB-activating signals emanate from cell surface receptors for inflammatory cytokines (such as TNF-α, IL-1, or LPS), growth factors, lymphokines, reactive oxygen intermediates, viral infection, and B or T cell activation [5]. NF-κB factors are homo- and heterodimeric transcription factors that belong to the Rel family; this family is composed of five homologous subunits in mammals: NF-κB1 (p50 and its precursor p105), NF-κB2 (p52 and its precursor p100), RelA/p65, RelB and c-Rel. Biological activity mediated by NF-κB occurs through two main signalling pathways, the classical and the alternative pathways, the former being specifically induced in response to TNF-α, whereas the latter is induced by other specific stimuli [6]–[8]. In the classical pathway, the inactive NF-κB complexes are sequestered in the cytoplasm through interaction with specific inhibitors, the IκB proteins (IκBα, IκBβ, especially). Following stimulation by TNF-α, activation of the IκB kinase (IKK) complex leads to the degradation of IκB proteins and subsequent release of mainly RelA/p50 dimers, which subsequently translocate into the nucleus where they bind to specific sites within the promoter of specific target genes. Based on studies on fibroblasts and B cell maturation, biological activity mediated by NF-κB may also occur through alternative pathway involving the activation of IKKα (IKK1) and the activation of RelB/p52 and RelB/p50 dimers [9]–[11].

Endothelial dysfunction has been implicated in the pathogenesis of diverse pathologic processes ranging from vascular diseases to cancer and metastasis. TNF-α mediated endothelial changes are relevant to acute inflammation, a highly complex biologic cascade-involving cytokines (such as IL6), chemokines IL-8 and MCP-1, and the induction of adhesion molecules (VCAM, ICAM and E selectin) [12]–[17] that recruit and activate granulocytes, monocytes/macrophages, and lymphocytes at the damaged tissue site. NF-κB often plays a major role in these pathways [17]–[18]. The diversity of the vascular bed is crucially determined by the type of endothelial cells which can be divided into microvascular and macrovascular endothelial cells. Although many reports have addressed the biological responses of human macrovascular endothelial cells (HUVEC) to TNF-α[14]–[15], [19], the detailed dissection of the coordinated expression of TNF-α-inducible genes *via* NF-κB has not been performed in human microvascular endothelial cells (HMEC).

In this study, we have determined the expression profile of 55 selected key genes in TNF-α-stimulated HMEC, that are all known as being regulated by NF-κB in other cell types in response to various stress responses [20]-[21]. HMEC were chosen, in addition to HUVEC which have been largely used in the past, because they originate from the microcirculation, their varying thresholds to cytokines have been previously described and they are often used in tumor angiogenesis studies. Using real-time reverse transcription (RT)-PCR, we demonstrated that 36.4 % (20/55) genes are markedly up-regulated (>5-fold) by TNF-α in a dose- and time-dependent pathway in HMEC. Using two NF-κB inhibitors and a direct approach of RelA expression knock-down by RNAi, we demonstrated that the gene signature observed in TNF-α stimulated HMEC is NF-κB-dependent. Intracellular signalling pathways triggered by TNF-α leading to numerous tumors have been partially delineated, with NF-κB playing an important role especially in breast cancer cells [20]–[25]. However, the stroma of a solid tumor is vital for its survival, and key components in this respect are the blood vessels. We further demonstrated that tumors producing high amount of TNF-α could have similar dysregulated NF-κB endothelial pathways.

## Materials and Methods

### Cell culture experiments

Human microvascular endothelial cells (HMEC) from derma (ATCC, Molsheim, France) were cultured in MCDB 131 medium (Gibco) supplemented with 10 % FBS, hydrocortisone (1 µg/ml; Sigma), epidermal growth factor (0.5%; Sigma), glutamine 2 mM, 100 U/ml penicillin and 100 µg/ml streptomycin. Human umbilical vein endothelial cells (HUVEC) were isolated with Collagenase I [26]. Cells were cultured on 0.2% gelatin-coated dishes in Medium 199 (M199) supplemented with 20% FBS, 2 mM glutamine, 100 µg/ml streptomycin, 15 mM of HEPES, and 0.4% ECGS (Promocell, Heidelberg, Deutschland). Second passaged cells and mix of three cords were used for all experiments. Cell cultures were incubated at 37°C in a humid atmosphere of 5 % CO2-95 % air. Once cells had grown to 80 % confluence, TNF-α (R&D Systems; Minneapolis, MN) was added into the medium at the indicated concentrations (1–80 ng/ml). The NF-κB inhibitors, Bay 11–7082 (10 µM; Calbiochem) [27] and the IκKβ kinase (IKK-2) inhibitor V (3,5-bis-trifluoromethyl-phenyl)5-cloro-2-hydroxybenzamide, Calbiochem) [28] were added in some experiences.

### Cell transfections

Subconfluent HMEC were transfected in OPTI-MEM medium (Gibco) with Fugene (4 µg per P30 flask; Roche) containing a construct of firefly luciferase ligated to a NF-κB -sensitive promoter of the gene [29]. Twenty hours later, cells were incubated with Bay 11–7082 (10 µM) and stimulated with TNF-α (40 ng/ml) for 4 h in the presence of the inhibitor, before they were harvested; pellets were analyzed for luciferase activity. Results are given as firefly luciferase activity as compared to TNF stimulation

#### Transient of siRNA

TNFR1 and TNFR2 were silenced using a specific siRNA mix containing three different siRNAs provided by Santa Cruz Biotechnology, as previously described [30]. As a control, siRNA with a nonsense sequence was usedg. Briefly, 10 µM of siRNA was added to the transfection reagent (Oligofectamine, InVitrogen; 1.25 µg) and subsequently transferred to culture plates P100 flask with 4×10^5^/flask HMEC. Then, 24 hours after transfection, cells were stimulated with TNF-α for 4 h and the specific experiment was performed.

#### Plasmid constructs

pTrip-shRNA RelA was generated by subcloning an oligonucleotide designed to target RelA under control of the H1 promoter into pTRIP-ΔU3-MND-GFP lentiviral vector [31]. pTRIP-shRNA control was generated following the same approach using a scrambled control oligonucleotide. Sequences are available upon request.

#### Lentiviral infection

Production of infectious recombinant lentiviruses was performed by transient transfection of 293T cells, as previously described [31]. For infections, 10^5^ cells in 35-mm dishes were transduced with 5000 ng/ml of p24 (HIV-1 capsid protein). 48 h later, cells were washed and fresh medium was added. The culture was then continued as described above.

### Electrophoretic Mobility Shift Assays (EMSA)

Nuclear extracts were prepared and analyzed for DNA binding activity using the HIV-LTR tandem κB oligonucleotide as κB probe [32]. For supershift assays, nuclear extracts were incubated with specific antibodies for 30 min on ice before incubation with the labeled probe. Anti-RelA, RelB, cRel, and p105/p50 supershifting antibodies were purchased from Santa Cruz Biotechnology.

### Western blotting and Antibodies

Cells were cultured in 100 mm dishes (Falcon) and harvested in lysis buffer (RIPA: PBS, 1% Nonidet P-40, 0.5% sodium deoxycholate, 0.1% SDS and protease inhibitors; Santa Cruz). After 30 min incubation on ice, lysates were cleared by centrifugation (12000 rpm, 10 minutes). Samples containing 50 µg of total protein from lysates were separated in an SDS/ polyacrylamide gel and transferred onto a nitrocellulose membrane. The rinsed and blocked membrane was then incubated with mouse antibodies against ICAM-1 (Santa Cruz biotechnologies, Inc, Santa Cruz, CA; 1/500), goat anti-IL-1β (R&D 1/500) or anti-GAPDH (Santa Cruz, 1/500), anti-rabbit polyclonal antibodies against RelA, RelB, p50/NF-κB1 or p52/NF-κB2 [32] (1∶1000; provided by R Weil and A Israel, Institute Pasteur, Paris), at room temperature for 1 hour. The blot was washed and probed with anti-mouse, -goat, or -rabbit antibodies conjugated with horseradish peroxidase (1∶10000; Dako, Glostrup, Denmark) at room temperature for 1 hour. The blot was washed and developed with ECL (Amersham biosciences, Orsay, France).

### Patients

To investigate the *in vivo* inter-relationships between mRNA levels of genes of interest, we analyzed 96 (48 ERα positive and 48 ERα negative) breast tumors excised from women at our institution (Centre René Huguenin, St Cloud, France). Samples containing more than 70% of tumor cells were considered suitable for this study. Immediately following surgery, the tumor samples were placed in liquid nitrogen until RNA extraction. The patients met the following criteria: primary unilateral non metastatic breast carcinoma; complete clinical, histological and biological information available; no radiotherapy or chemotherapy before surgery; and full follow-up at our institution. Estrogen receptor alpha status was determined at the protein level by biochemical method (enzymatic immunoassay) and confirmed by ERα real-time quantitative RT-PCR assay [33]. The malignancy of infiltrating carcinomas was scored according to Scarff Bloom and Richardson's histoprognostic system [34]. The characteristics of these patients are shown in **Table 1**.

**Table 1:** Characteristics of the 48 ERα-positive and the 48 ERα-negative breast tumors.

	ERα-positive breast tumors			ERα-negative breast tumors		
	Number of patients	Number of relapses (%)	*p-*value^a^	Number of patients	Number of relapses (%)	*p*-value^a^
***Age***						
≤70	27	14 (51.9)	NS	40	17 (42.5)	NS
>70	21	10 (47.6)	(0.39)	8	2 (25.0)	(0.51)
***SBR histological grade*** b						
I + II	38	16 (42.1)	NS	6	5 (83.3)	NS
III	10	8 (80.0)	(0.13)	39	14 (35.9)	(0.058)
***Lymph node status***						
≤3	32	13 (40.6)	**0.023**	41	16 (39.0)	NS
>3	16	11 (68.7)		6	3 (50.0)	(0.96)
***Macroscopic tumor size***						
≤30 mm	32	14 (43.8)	NS	27	13 (48.1)	NS
>30 mm	16	10 (62.5)	(0.076)	20	6 (30.0)	(0.15)

a Log-rank test. NS: not significant.

b Scarff Bloom Richardson classification.

### RNA extraction

RNA was isolated using TRIzol reagent according to the manufacturer's instructions (Invitrogen, Cergy Pontoise, France). The quality of the RNA samples was determined by electrophoresis through agarose gels and staining with ethidium bromide, and the 18S and 28S RNA bands were visualized under ultraviolet light.

### Real-time quantitative-PCR

Transcript quantification of NF-κB selected genes was performed as previously described [35], using the TATA box-binding protein (TBP) as the endogenous RNA control; each sample was normalized on the basis of its TBP content. Primers for TBP and the 55 target genes (**Table 2**) were chosen with the assistance of the Oligo 5.0 computer program (National Biosciences, Plymouth, MN). We searched the dbEST and nr databases to confirm the total gene specificity of the nucleotide sequences chosen as primers, and the absence of single nucleotide polymorphisms. In particular, the primer pairs were selected to be unique relative to the sequences of closely related family member genes or of the corresponding retropseudogenes. To avoid amplification of contaminating genomic DNA, one of the two primers was placed at the junction between two exons, if possible. In general, amplicons were between 60 and 120 nucleotides long. Gel electrophoresis was used to verify the specificity of PCR amplicons. For each primer pair we performed no-template control (NTC) and no-reverse-transcriptase control (RT-negative) assays, which produced negligible signals (usually >40 in Ct values), suggesting that primer-dimer formation and genomic DNA contamination effects were negligible. The cDNA synthesis and PCR conditions have been described in detail elsewhere [35]. Results are expressed as N-fold differences in target gene expression relative to TBP gene expression.

**Table 2:** List of the 55 selected genes tested.

Gene symbols	Protein symbols	Gene name	Chromosome location	Genbank accession number
*NFKB genes (n = 10)*				
*NFKB1*	p50, p105	Nuclear factor of kappa light polypeptide gene enhancer in B-cells 1	4q24	NM_003998
*NFKB2*	p52, p100	Nuclear factor of kappa light polypeptide gene enhancer in B-cells 2	10q24	NM_002502
REL	C-Rel	v-rel reticuloendotheliosis viral oncogene homolog	2p13-p12	NM_002908
*RELA*	p65, NFKB3	v-rel reticuloendotheliosis viral oncogene homolog A (p65)	11q13	NM_021975
RELB	I-REL	v-rel reticuloendotheliosis viral oncogene homolog B	19q13.32	NM_006509
*CHUK*	IκKα	Conserved helix-loop-helix ubiquitous kinase	10q24-q25	NM_001278
*IKBKB*	IκKβ	Inhibitor of kappa light polypeptide gene enhancer in B-cells, kinase beta	8p11.2	NM_001556
*IKBKG*	IκKγ, Nemo	Inhibitor of kappa light polypeptide gene enhancer in B-cells, kinase gamma	Xq28	NM_003639
*NFKBIA*	IκBα	Nuclear factor of kappa light polypeptide gene enhancer in B-cells inhibitor, alpha	14q13	NM_020529
*NFKBIB*	IκBβ	Nuclear factor of kappa light polypeptide gene enhancer in B-cells inhibitor, beta	19q13.1	NM_002503
*Apoptosis (n = 12)*				
*BCL2A1*	BFL1/A1	Baculoviral IAP repeat-containing 2	15q24.3	NM_004049
*GADD45B*		Growth arrest and DNA-damage-inducible, beta	19p13.3	NM_015675
*TNFRSF10B*	TRAILR2	Tumor necrosis factor receptor superfamily, member 10b	8p22-p21	NM_003842
*FASLG*	TNFSF6	Fas ligand (TNF superfamily, member 6)	1q23	NM_000639
*BIRC4*	XIAP	Baculoviral IAP repeat-containing 4	Xq25	NM_001167
*TNFAIP3*	A20	Tumor necrosis factor, alpha-induced protein 3	6q23	NM_006290
*TRAF2*		TNF receptor-associated factor 2	9q34	NM_021138
*IER3S*		Immediate early response 3, large transcript	6p21.3	NM_003897
*IER3L*		Immediate early response 3, short transcript	6p21.3	NM_052815
*BIRC2*	c-IAP1	Baculoviral IAP repeat-containing 2	11q22	NM_001166
*MCL1S*		Myeloid cell leukemia sequence 1 (BCL2-related), short transcript	1q21	NM_182763
*MCL1L*		Myeloid cell leukemia sequence 1 (BCL2-related), large transcript	1q21	NM_021960
*Immune response (n = 12)*				
*IL1A*		Interleukin 1, alpha	2q14	NM_000575
*IL1B*		Interleukin 1, beta	2q14	NM_000576
*IL6*		Interleukin 6 (interferon, beta 2)	7p21	NM_000600
*IL12B*		Interleukin 12B (natural killer cell stimulatory factor 2)	5q31.1-q33.1	NM_002187
*CCL2*	MCP-1	Chemokine (C-C motif) ligand 2	17q11.2-q21.1	NM_002982
*TNFRSF11A*	RANK	Tumor necrosis factor receptor superfamily, member 11a, activator of NFKB	18q22.1	NM_003839
*TNFSF11*	RANKL	Tumor necrosis factor (ligand) superfamily, member 11	13q14	NM_003701
*TNF*		Tumor necrosis factor (TNF superfamily, member 2)	6p21.3	NM_000594
*TNFRSF1A*	TNFR1	Tumor necrosis factor receptor superfamily, member 1A	12p13.2	NM_001065
*TNFRSF1B*	TNFR2	Tumor necrosis factor receptor superfamily, member 1B	1p36.3-p36.2	NM_001066
*CD40*		CD40 antigen (TNF receptor superfamily member 5)	20q12-q13.1	NM_001250
*CD40LG*		CD40 ligand (TNF superfamily, member 5, hyper-IgM syndrome)	Xq26	NM_000074
*Cell Proliferation (n = 7)*				
*CSF1*	MCSF	Colony stimulating factor 1 (macrophage)	1p21-p13	NM_000757
*CSF1R*		Colony stimulating factor 1 receptor, v-fms oncogene homolog	5q33-q35	NM_005211
*CSF2*	GMCSF	Colony stimulating factor 2 (granulocyte-macrophage)	5q31.1	NM_000758
*CCND1*		Cyclin D1 (PRAD1 : parathyroid adenomatosis 1)	11q13	NM_053056
*CCND2*		Cyclin D2	12p13	NM_001759
*CCND3*		Cyclin D3	6p21.3	NM_001760
*CCNG1*		Cyclin G1	5q32-q34	NM_004060
*Tumor progression (n = 9)*				
*MMP9*		Matrix metalloproteinase 9 (gelatinase B, 92kDa type IV collagenase)	20q11.2-q13.1	NM_004994
*MMP11*		Matrix metalloproteinase 11 (stromelysin 3)	22q11.23	NM_005931
*PLAU*	UPA	Plasminogen activator, urokinase	10q24	NM_002658
*CXCR4*		Chemokine (C-X-C motif) receptor 4	2q21	NM_003467
*CXCL12*	SDF1	Chemokine (C-X-C motif) ligand 12 (stromal cell-derived factor 1)	10q11.1	NM_000609
*ICAM1*		Intercellular adhesion molecule 1 (CD54), human rhinovirus receptor	19p13.3-p13.2	NM_000201
*VCAM1*		Vascular cell adhesion molecule 1	1p32-p31	NM_001078
*SELE*	ELAM1	Selectin E (endothelial adhesion molecule 1)	1q22-q25	NM_000450
*TNC*	HXB	Tenascin C (hexabrachion)	9q33	NM_002160
*Angiogenesis (n = 4)*				
*IL8*		Interleukin 8	4q13-q21	NM_000584
*CXCL1*	GRO1	Chemokine (C-X-C motif) ligand 1 (melanoma growth stimulating activity, alpha)	4q21	NM_001511
*VEGF*	VEGFA	Vascular endothelial growth factor	6p12	NM_003376
PTGS2	COX2	Prostaglandin-endoperoxide synthetase 2	1q25.2-q25.3	NM_000963
*Divers (n = 1)*				
EGR1		Early growth response 1	5q31.1	NM_001964

### Statistical analysis

Statistical analysis of *in vitro* experiments was performed by analysis of mean with the Student t test. The terms n = x is used to indicate the number of independent experiments performed. Correlations between *TNF* and the gene target expressions were tested using the non parametric Spearman rank correlation test (which compared continuous variables). Differences were judged significant at confidence levels greater than 95% (p<0.05).

## Results

### Identification of TNFα-dependent NF-κB-associated genes in microvascular endothelial cells

To determine the expression pattern of NF-κB-associated genes in response to TNF-α in microvascular endothelial cells, we have performed real-time RT-PCR in HMEC stimulated by TNF-α for 4 h. Our preliminary kinetic analysis on gene candidates (such as E-selectin and IL-8) suggested an induction at 2–4 h time point induction. Among the 55 genes tested (**Table 2**), only one gene, *IL12B*, presented detectable but not reliably quantifiable mRNA expression (Ct>35). Twenty (37 %) of the other 54 genes were up-regulated by 5-fold or more (until 1200-fold) in HMEC stimulated with TNF-α as compared to untreated cells (**Table 3**). These robust selection criteria (a cut-off of 5-fold expression difference in HMEC stimulated with TNF-α) ensure identification of genes with marked interest.

**Table 3:** Effect of tumor necrosis factor (TNF-α treatment on selected *NF-*κ*B* gene expression in HMEC cells.

Gene	*Mean* a *+/− SEM*
***TNF***	1156+/− 336
***SELE***	1022+/− 102
***VCAM***	896+/− 246
***CSF2/GMCSF***	762+/− 89
***IL8***	726+/− 91
***ICAM***	357+/− 103
***CCL2/MCP1***	75+/− 8,2
***CXCL1/GRO1***	71+/− 8,3
***RELB***	63+/− 11,4
***TNFAIP3/A20***	49+/− 7,5
***IL1A***	31+/− 0,7
***IL1B***	26+/− 0,5
***BCL2A1***	21+/− 2,3
***NFKB2***	20+/− 1,9
***NFKBIA***	18+/− 0,8
***CSF1***	14+/− 1,1
***NFKB1***	8,7+/− 0,4
***CD40***	5,3+/− 1,1
***IL6***	5,3+/− 0,6
***TNFRSF10B***	5,1+/− 0,1
***MKI67***	0,9+/− 0,1
***ESR1/ERα***	NQ^b^

a Ratio of the mRNA content in stimulated cells for 4 hours by TNF-α to the mRNA content in the unstimulated cells was calculated. This time was chosen from preliminary kinetic analysis on gene candidates (such as E-selectin and IL-8) (see also Table 4). Values shown are calculated from three independent experiments, each experiment in duplicate.

b NQ, not quantifiable: very low levels of target gene mRNA that were only detectable, but not quantifiable, by means of the real-time quantitative RT-PCR assay (Ct values >35).

The 20 identified genes belong to several groups according to their biological function: adhesion molecules (*SELE* encoding for E-selectin, *VCAM*, *ICAM*, and *CD40),* chemokines (*CSF2/GMCSF, IL8, CCL2/MCP1, CXCL1/GRO1* and *CSF1)*, and cytokines (*TNF*, *IL1A*, *IL1B* and *IL6)* (**Table 3**). Both *TNFR1* and *TNFR2* were expressed at a similar level in endothelial cells, and were not regulated by TNF-α (*not shown*). Interestingly, three anti-apoptotic proteins, TNFAIP3/A20, BCL2A1 and TNFRSF10B/TRAILR2/DR5 were markedly up-regulated. *BIRC2* was moderately up-regulated (4.4 fold), while other genes involved in apoptosis, such as *FASL, TRAF2, MCL1* and *BIRC4* were not (*not shown*).

The expression of several components of the NF-κB signalling cascades was also up-regulated in TNF-α stimulated HMEC, as compared to untreated cells (**Table 3**). *RELB* (gene encoding RelB) was strongly up-regulated (63*-*fold induction). In addition *NFKB2, NFKB1* and *NFKBIA*, which respectively encode NF-κB2 (p52/p100), NF-κB1 (p50/p105) and NF-κBIA/inhibitor IκBα were more moderately up-regulated (10 to 20-fold). In contrast, *RELA* (encoding RelA/p65), *REL* (encoding c-Rel) and *NFKB1B* were increased only 2 to 3-fold (*not shown*). Genes encoding kinases, *CHUK* (IκKα), *IKBKB* (IκKβ) and *IKBKG* (NEMO or IκKγ) were not up-regulated (*not shown*).

In contrast, TNF-α did not modify the expression of a few set of genes known to be regulated by TNFα in tumor cells (such as MMP9). In the same set of samples, TNF-α did not modify the expression of MKI67 gene encoding the proliferation-related Ki-67 antigen cell proliferation marker.

**Figure 1:**
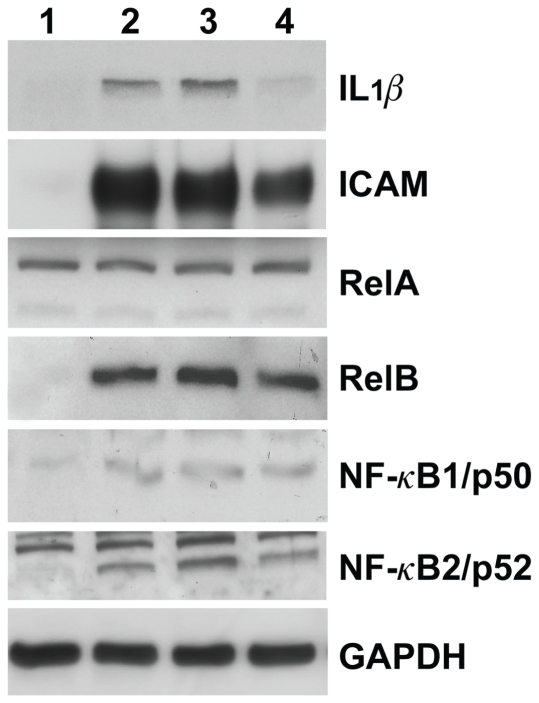
Western blot analyses of IL-1β, ICAM, RelA, RelB, NF-κB1/p50 and NF-κB2/p52 proteins in TNF-α stimulated HMEC. HMEC cells were incubated for 4 h with 4–40 ng/ml TNF-α. Whole cell lysates were prepared and subjected to Western blotting using anti-ICAM, anti-IL-1β anti-RelA, anti-RelB, anti-NF-κB1/p50 or anti-NF-κB2/p52 antibodies, as indicated in *Materials and Methods*. As loading controls, total proteins were also analyzed with anti-GAPDH. Each lane contains 50 µg of cellular protein. Lanes 1–4 correspond to control endothelial cells (lane 1), endothelial cells stimulated with 40 ng/ml (lanes 2 and 3) or 4 ng/ml (lane 4) TNF-α. Results were similar in two independent experiments.

To confirm that the increase of mRNA expression correlates with induction of protein expression, cell lysates from TNF-α stimulated HMEC were subjected to western immunoblotting using antibodies directed against ICAM, IL-1β or several proteins of the NF-κB and IκB families. As shown in **Fig. 1**, treatment of HMEC with TNF-α strongly up-regulated the expression of IL-1β ICAM and RelB. TNFα also increased NF-κB1/p50 and NF-κB2/p52 expression in endothelial cells, whereas modulation of RelA protein level was not observed, in agreement with findings at the mRNA levels.

### TNF-α modulates NF-κB - associated genes in a dose- and time-dependent manner

Expression of the NF-κB - associated genes was up-regulated with TNF-α in a dose-dependent manner (1–80 ng/ml) in HMEC with the same profile of response (maximum degree of response for 40 ng/ml of TNF-α) (**Table 4** **).** Ten of these genes were up-regulated more than 10-fold at 0.2 ng/ml, as compared to controls, with *VCAM, CSF2/GMCSF* and *IL8* being stimulated more than 100-fold.

**Table 4:** Dose-dependent effect of TNF-α treatment (0–80 ng/ml) on selected NFkB gene expression in HMEC cells.

	TNF-α concentration					
Gene	0.2 ng/ml	1 ng/ml	2.5 ng/ml	10 ng/ml	40 ng/ml	80 ng/ml
***TNF***	53^a^	225	559	769	1241	1210
***SELE***	13	65	359	609	1016	1029
***VCAM***	113	215	610	832	958	937
***CSF2/GMCSF***	140	314	443	463	714	583
***IL8***	179	520	691	926	1123	918
***ICAM***	16	63	114	123	155	139
***CCL2/MCP1***	16	42	53	55	70	56
***CXCL1/GRO1***	40	67	76	69	92	71
***RELB***	30	42	60	55	63	52
***TNFAIP3/A20***	15	26	25	32	44	32
***IL1A***	4.6	13	22	25	37	36
***IL1B***	3.2	7.8	14	14	21	17
***BCL2A1***	3.3	6.9	14.2	13.8	21	13
***NFKB2***	6.2	11.4	16	17	20	17
***NFKBIA***	7.6	10	12	12	19	13
***CSF1***	4.3	7.8	11	11	13	10
***NFKB1***	2.4	3.2	3.7	4.1	5.3	4.2
***CD40***	1.4	1.2	1.7	1.8	5.1	2.5
***IL6***	1.1	1.9	2.9	3.1	5.7	3.1
***TNFRSF10B***	2.3	3.2	3.7	3.8	5.1	3.8

a Ratio of the mRNA content in cells stimulated with TNF-α for 4 h to the mRNA content in the unstimulated cells.

We also investigated the kinetics of TNF-α-induced mRNA expression. HMEC were treated with TNF-α (40 ng/ml) at different time periods (0 to 72 h). We observed the same profile of response for all genes, with a maximum induction always observed at 4 hours of TNF-α treatment (**Table 5**), associated to a marked decreased at 24 and 72 hours.

**Table 5:** Kinetics of selected NFkB gene expression in TNF-α treated HMEC.

	Time							
Gene	2 minutes	8 minutes	15 minutes	30 minutes	1 hour	4 hours	24 hours	72 hours
***TNF***	1.2 a	2.0	2.6	5.1	12	1241	335	2.7
***SELE***	0,9	1,0	1,2	1,2	1,3	1016	58	NQ
***VCAM***	1,0	1.1	1,2	1.8	27	958	88	6.7
***CSF2/GMCSF***	1.5	1.2	1.7	19	21	714	219	6.1
***IL8***	1.5	4.5	11	57	17	1123	331	11
***ICAM***	0.7	0.8	0.7	0.8	14	155	15	6.5
***CCL2/MCP1***	0.7	0.7	0.9	1.6	8.6	70	20	3.7
***CXCL1/GRO1***	1.1	3.2	6.8	11.4	4.2	92	23	2.9
***RELB***	1.0	1.0	1.0	1.0	25	63	30	18.5
***TNFAIP3/A20***	0.9	0.9	0.9	2.5	1.7	44	26	3.7
***IL1A***	0.6	0.6	0.6	0.7	1.0	37	7.6	0.7
***IL1B***	0.6	0.7	0.6	1.2	3.1	21	15	1.1
***BCL2A1***	1.1	1.0	1.1	1.1	3.3	21	16	0.9
***NFKB2***	1.6	1.0	1.6	1.4	8.8	20	12.1	5.7
***NFKBIA***	0.5	0.6	0.8	1.3	1.2	19	2.0	1.2
***CSF1***	1.0	0.8	1.0	0.9	3.5	13	5.3	2.9
***NFKB1***	1.0	0.9	1.1	1.0	1.7	5.0	2.8	1.5
***CD40***	0.6	0.4	0.6	0.7	1.6	4.7	1.7	1.8
***IL6***	0.7	0.9	1.0	2.6	1.7	5.4	3.3	1.9
***TNFRSF10B***	1.0	0.8	1.0	0.9	1.3	5.1	2.1	1.3

a Ratio of the mRNA content in cells stimulated with TNF-α (40 ng/ml) for 2, 8, 15, 30 min or 1, 4, 24 and 72 h to the mRNA content in the control cell.

### Quantitative and qualitative differences in the expression of the NF-κB-associated genes between microvascular and macrovascular endothelial cells

**Figure 2:**
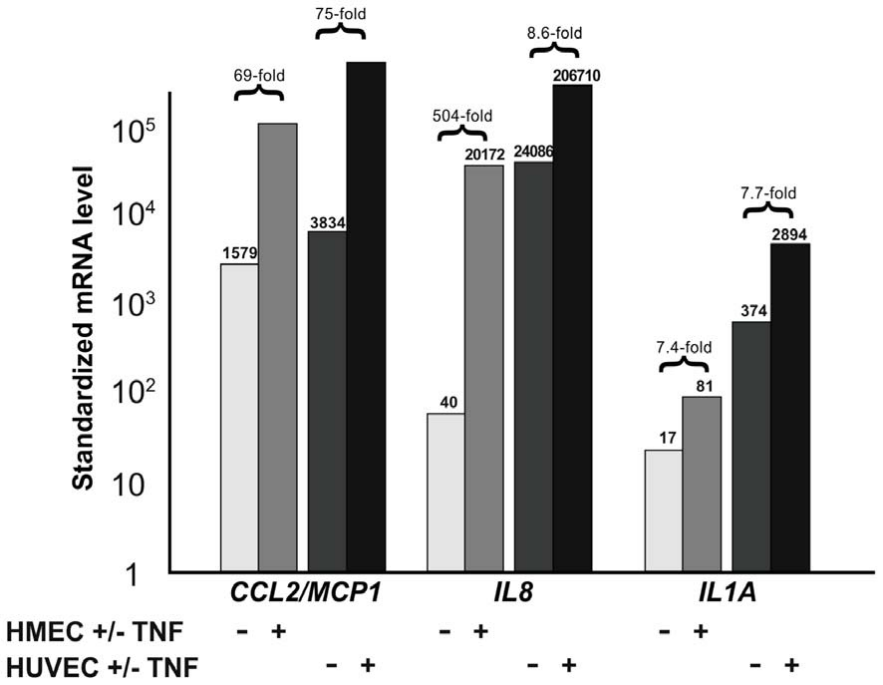
mRNA expression of CCL2/MCP1, IL8 and IL1A in TNF-α-stimulated and unstimulated HUVEC and HMEC cells. For each gene, mRNA levels were normalized such that the value for the “basal mRNA level” (smallest amount of target gene mRNA quantifiable, Ct = 35) was 1.

Quantitative RT-PCR was used to compare the expression of NF-κB associated genes in TNF-α stimulated HUVEC and HMEC (40 ng/ml for 4 h). As shown in **Table 6**, TNF-α up-regulated the expression of the same 20 NF-κB associated genes on the two classes of endothelial cells, except for *CD40*, *IL6* and *TNFRSF10B* that are hardly induced in HUVEC. Interestingly, for *VCAM*, *ICAM*, *IL8*, *CSF2*, *CXCL1/GRO1*, *RELB, IL6* and *TNFRSF10B* expression, the fold induction was much higher in HMEC, as compared to HUVEC. However, these results were probably due to a lower basal expression level in HMEC compared to HUVEC (**Table 6**). **Fig. 2** shows the mRNA levels of three characteristic genes (*CCL2/MCP1*, *IL8* and *IL1A*) in TNF-α-stimulated HUVEC and HMEC cells. Finally, it is interesting to note that among the 35 genes whose expression was not up-regulated (>5-fold) in stimulated HMEC, no gene was up-regulated in HUVEC (>5-fold) (not shown).

**Table 6:** TNF-α up-regulates the expression of the same 20 NF-κB associated genes in HMEC and HUVEC.

	HMEC cells			HUVEC cells		
Gene	Without TNF-α	With TNF-α	Fold change	Without TNF-α	With TNF-α	Fold change
***TNF***	1	411	**411**	1	198	**198**
***SELE***	1	743	**743**	23	20123	**875**
***VCAM***	1	1314	**1314**	553	150276	**272**
***CSF2/GMCSF***	1.3	1027	**790**	23	3577	**155**
***IL8***	40	20172	**504**	24086	206710	**8.6**
***ICAM***	278	89187	**321**	1987	152666	**77**
***CCL2/MCP1***	1579	108418	**69**	3834	288180	**75**
***CXCL1/GRO1***	101	12752	**126**	11350	56063	**4.9**
***RELB***	145	10712	**74**	827	6903	**8.4**
***TNFAIP3/A20***	643	37685	**59**	3697	84782	**23**
***IL1A***	11	81	**7.4**	374	2894	**7.7**
***IL1B***	59	689	**12**	53	664	**13**
***BCL2A1***	1	27	**27**	19	733	**39**
***NFKB2***	269	6729	**25**	1191	8898	**7.5**
***NFKBIA***	1682	35376	**21**	3144	23936	**7.6**
***CSF1***	540	7385	**14**	2252	24483	**11**
***NFKB1***	1328	12972	**9.8**	2503	12581	**5.0**
***CD40***	150	944	**6.3**	1216	2426	2.0
***IL6***	49	384	**7.8**	290	902	3.1
***TNFRSF10B***	1186	6357	**5.3**	11253	21756	1.9

Cells were incubated in the presence of TNF-α (40 ng/ml) for 4 h. Gene expression was measured by RT-PCR, as described in *Materials and Methods*. For each gene, mRNA levels were normalized such that the value for the “basal mRNA level” (smallest amount of target gene mRNA quantifiable, Ct = 35) was 1. Ratio of the mRNA content in stimulated cells *versus* unstimulated cell mRNA content was calculated. Results are expressed as the mean of two independent experiments.

### TNFR1 and, to a lesser extent, TNFR2 stimulate the NF-κB -- associated genes

We then analysed the mechanisms of the regulation by TNF-α observed in endothelial cells first using silencing of TNF receptors. Since TNFR1 (or TNF-Rp55) and TNFR2 (or TNF-Rp75) are both present in endothelial cells, we silenced the expression of each receptor by RNA interference in HMEC. In the presence of TNF-α, the expression of TNFR1 was reduced from 45 % in siRNA TNFR1-transfected cells, as compared to siRNA control (**Table 7**). In these cells, the expression of twelve NF-κB associated genes including *VCAM, ICAM, CSF2/GMCSF* and *CSF1,* the cytokines *TNF, IL8, IL1A, IL1B* and *IL6*, *TNFAIP3*, *BCL2A1* and *NFKB1* was inhibited more than 30 %, as compared to TNF-α stimulated siRNA control-transfected cells (**Table 7**). In contrast, *TNFR2* expression was not reduced in siRNA TNFR1-transfected cells, suggesting that the decrease of TNFR1 expression was receptor specific. We further tested the putative involvement of TNFR2 in TNF-α response in endothelial cells. The expression of *TNFR2,* but not *TNFR1*, was reduced from 77 % in HMEC transfected with siRNA TNFR2. Only 4 genes including *CSF2/GMCSF*, *IL1B, TNFRSF10B* and *TNF* were inhibited more than 15 % (28 %, 26 %, 22 % and 17 %, respectively) when *TNFR2* was silenced (**Table 7**). Altogether, these results indicate that TNFR1 and, to a lesser extent, TNFR2 are involved in the control of the NF-κB associated gene signature in TNF-α stimulated endothelial cells.

**Table 7:** Inhibitory effect of SiRNA TNFR on TNF-α-induced genes in HMEC endothelial cells.

Gene	Control SiRNA +TNF-α	SiRNA TNFR1 + TNF-α	% inhibition *via* R1	Control SiRNA +TNF-α	SiRNA TNFR2 + TNF-α	% inhibition *via* R2
**TNF R1**	1.7	0.94	**45**	0.95	0.89	**7**
***TNFR2***	1.41	1.56	**0**	10.3	2.43	**77**
***TNF***	>225	>158	**30**	185.5	155	**17**
***SELE***	>19	>9	**ND**	>65	>51	**ND**
***VCAM***	124	75.6	**40**	>153	>218	**ND**
***CSF2/GMCSF***	92	52.5	**43**	97	70	**28**
***IL8***	86	43.5	**50**	59.3	62	**0**
***ICAM***	288	171.8	**40**	113	101.5	**11**
***CCL2/MCP1***	162.7	122.3	**25**	61.8	59.4	**4**
***CXCL1/GRO1***	21.7	17	**22**	18.6	19	**0**
***RELB***	17.3	14.5	**16**	6.47	6.49	**0**
***TNFAIP3/A20***	12.6	8.4	**33**	10.1	10.7	**0**
***IL1A***	11.7	5	**57**	18.8	17.8	**6**
***IL1B***	81.4	39.7	**51**	25.5	19	**26**
***BCL2A1***	10.5	5.5	**48**	10	12	**0**
***NFKB2***	10.6	7.6	**28**	4.8	5	**0**
***NFKBIA***	8	5.8	**27**	4.43	4.78	**0**
***CSF1***	15.7	10.9	**30**	7.7	7.9	**0**
***NFKB1***	6.4	4.5	**30**	3.5	3.5	**0**
***CD40***	3.3	2.5	**23**	3.3	3.1	**5**
***IL6***	8.7	5.2	**40**	5.13	5.43	**0**
***TNFRSF10B***	3.7	2.7	**26**	3.56	2.8	**22**

a Results are expressed as ratio (in percentage) of the mRNA content in siRNA TNFR-transfected cells stimulated with TNF-α (40 ng/ml) for 4 h to the mRNA content in unstimulated cells. After transfection with siTNFR1 or siTNFR2 RNAs, TNFR1 and TNFR2 expression were strongly inhibited (45 % and 77 %, respectively). Results are expressed as the mean of two different independent experiments.

### NF-κB activation is involved in TNF-α-induced gene expression in endothelial cells

We next examined the effects of chemical inhibition of IκB phosphorylation and IkappaB kinase activity on gene expression in TNF-stimulated HMEC **(** **Table 8** **)**. Bay 11–7082, an inhibitor IκB phosphorylation [27] was first tested on NF-κB activity in endothelial cells using a luciferase reporter gene whose activity depends on a κB element. Luciferase activity was decreased by 99 % after addition of Bay 11–7082 (10 µM) on TNF-α treated endothelial cells (*data not shown*). Most importantly, the addition of Bay 11–7082 resulted in decreased expression of all selected genes in TNF-α treated HMEC (**Table 8**). As a control, TNFR receptor expression which was not changed by TNF-α stimulation was insensitive to Bay 11–7082. The addition of pharmacological IκB kinase (IKK2) inhibitor V [28] also inhibited the expression of *VCAM*, *ICAM*, *CCL2/MCP1* and *BCL2A1* and *IL1B*at 1–10 µM (**Table 8**); surprisingly, it activated *CSF2/GMCSF, TNFAIP3, IL1A* and *IL8.* Altogether these experiments using two different inhibitors suggest that canonical NF-κB signalling pathway is involved in TNF-κ-induced gene expression in endothelial cells.

**Table 8:** Inhibitory effects of Bay 11–7082 and IKK2 inhibitor V on TNF-α-induced genes in HMEC endothelial cells.

Gene	TNF-α	TNF-α + Bay 11-7082	TNF-α	IKK2+TNF-α 1 µM	IKK2+TNF-α10 µM
***TNF R1***	0.6	0.8	0.86	0.8	1
***TNFR2***	0.8	2.4	1.8	1.1	1
***TNF***	78	NE	34	19	*22*
***SELE***	9.5	1.6	4	4	3.7
***VCAM***	47.5	NE	24.8	24.9	7.3
***CSF2/GMCSF***	22.8	0.4	136	*286*	337
***IL8***	157	2.2	246	419	368
***ICAM***	332	1.1	450	284	261
***CCL2/MCP1***	85	0.9	162	114	42.3
***CXCL1/GRO1***	20	1	33.4	39.5	29.2
***RELB***	12.4	0.5	28	26.8	22.7
***TNFAIP3/A20***	11	2.4	17.6	26.6	29.6
***IL1A***	20.3	0.9	23.4	30.4	57
***IL1B***	15.6	1.5	29.8	25.6	16.3
***BCL2A1***	*2.6*	0.3	9	4.6	3.6
***NFKB2***	7.5	1	12.7	10.1	10
***NFKBIA***	5.5	0.6	6.3	6.8	7.7
***CSF1***	14.9	1.1	16.5	14.7	16.2
***NFKB1***	4.4	0.8	6	6	5.3
***CD40***	2.6	1.6	2.1	1.7	1.8
***IL6***	8	1.2	10.7	10.7	13.4
***TNFRSF10B***	3	2.2	4.3	3.6	4.4

Cells were pre-treated with pharmacological inhibitors of NF-κB, Bay 11–7082 10 µM or IKK2 inhibitor V (1 and 10 µM), for 30 min before incubation with 40 ng/ml TNF-α for 4 h. mRNA levels were normalized to unstimulated cells which values were set to 1.

**Figure 3:**
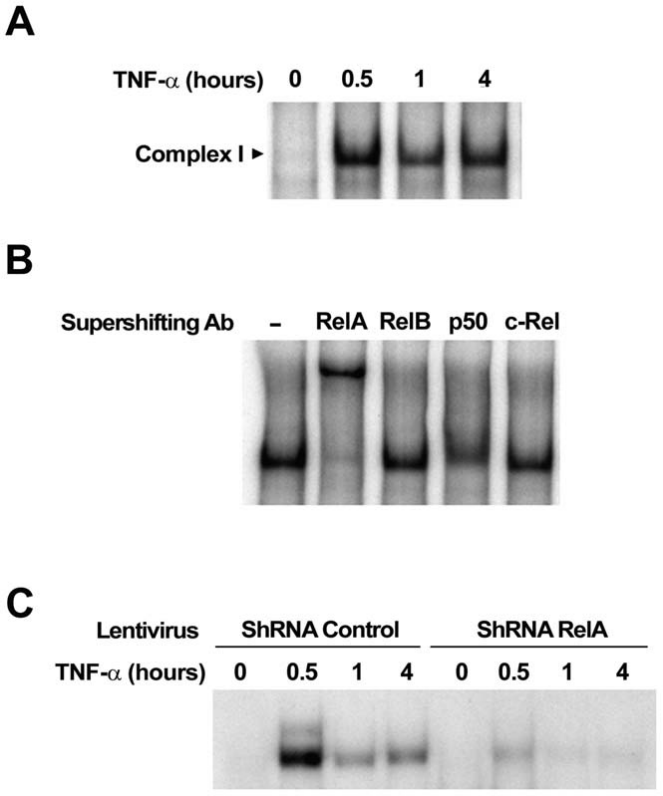
RelA knock-down by RNAi blocks TNFα-induced gene expression in HMEC cells. (A) Nuclear extracts from HMEC cells treated with TNF-α for the indicated periods of time were analyzed by EMSA using a ^32^P-labeled HIV-LTR tandem κB oligonucleotide as a probe. (B) For supershift, nuclear extracts from HMEC cells treated with TNFα for 4 hours were incubated with the indicated antibodies before incubation with the labeled probe. Complex I: RelA/p50. (C) Nuclear extracts from HMEC cells stably transduced with a lentivirus encoding a shRNA targeting either RelA or a scrambled control, and treated by TNFα for the indicated periods of time were analyzed by EMSA as described in (A).

To directly demonstrate the involvement of NF-κB in the gene signature induced by TNF-α in endothelial cells, we first evaluated TNFα-induced activation of NF-κB complexes in HMEC cells by EMSA. As shown in **Fig 3A**, TNF-α treatment in HMEC resulted in a characteristic biphasic induction of κB DNA binding (complex I). Supershift analysis of nuclear extracts stimulated for 4 h with TNF-α indicated that the NF-κB DNA-binding complexes consist of RelA-p50 dimers (**Fig 3B**).

We then used stable RNA interference knock-down to inhibit RelA activity in HMEC cells. We infected HMEC cells with either a lentivirus carrying a shRNA targeting RelA or a scrambled control. Whereas TNFα stimulation of scrambled control-infected HMEC cells induced RelA DNA binding with kinetics that parallel what is seen in non-infected cells, RelA expression knock-down almost completely abolished RelA-DNA binding activity (**Fig 3C**).

We then examined the mRNA expression levels of 16 genes by real time RT-PCR. The down-regulation of RelA activity clearly inhibited the expression of all of them except TNFRSF10B **(** **Table 9** **)**, thus indicating that RelA is required for the TNF-α-induced expression of these genes in HMEC cells.

**Table 9:** Effect of ShRNA RelA on TNF-α-induced genes in HMEC endothelial cells.

Gene	Control ShRNALuc +TNF-α	ShRNA RelA + TNF-α	% inhibition
***CCL2***	8.4	2.1	**74.8**
***CD40***	1.6	1	**41.4**
***CSF1***	3.2	2.2	**31.9**
***CSF2***	79.7	40.8	**48.8**
***CXCL1***	18.6	3.45	**81.4**
***ICAM***	17.7	2.1	**85.8**
***IL1A***	55.8	12.3	**77.9**
***IL1B***	44	4.1	**90.5**
***IL6***	17	0	**100**
***IL8***	135.9	10.4	**92.3**
***NFKB1***	3	1.2	**60.5**
***NFKB2***	11.5	6.2	**46**
***NFKBIA***	22.3	3.1	**85.8**
***RelB***	5.4	1.4	**74.6**
***TNFAIP3***	30	6.8	**77.1**
***TNFRSF10B***	2.4	2.4	**0**

For each gene and each sample, mRNA levels were normalized such that the value of the Control shRNALuc sample was 1.The inhibition by ShRNA RelA is expressed as pourcentage of the difference between control shRNA Luc vs shRNA RelA in cells stimulated with TNF-α (40 ng/ml) for 4 h. Genes were classified by alphabetical order.

### Positive correlation between the expression of TNF-α mRNA and of the 19 other identified putative TNF-inducible genes in 96 human breast tumors

TNF-α is highly expressed in tumors where it has been considered initially as a potent tumor cell killer and an anti-vascular cytokine at high doses [36]. However, low chronic doses of the cytokine are thought to be pro-angiogenic [4]. In order to test the involvement of TNF-α activation/endothelial dysfunction of the breast tumor vasculature, we further explored if the correlations identified in our ex vivo cellular model (endothelial cells) between the expression of TNF-α mRNA and the expression of 19 putative TNF-α- inducible NF-κB target genes were also observed *ex vivo* in human breast tumors.

This study was performed on a series of 96 breast tumors (48 ERα-positive and 48 ERα-negative tumors) (**Table 1**). We observed a positive correlation between *TNF* and 16 (84.2 %) of the 19 endothelial TNF-α-inducible genes in both ERα-negative and ERα-positive breast tumors (**Table 10**). The three remaining genes (i.e., *IL8, IL1A* and *IL6*) were significantly linked to *TNF* only in ERα-negative tumors. In the same set of 96 tumors, we also examined the expression of the *ESR1* gene, which encodes ERα as well as the proliferation-associated gene *MKI67*, which encodes the proliferation-related antigen Ki-67. We found no correlation between *TNF* and *ESR1* and *MKI67*, suggesting that the 19 genes were associated to TNF independently to hormonal status and cell proliferation.

**Table 10:** Relationship between expression of *TNF* and expression of the 19 others identified putative TNF-inducible genes in 48 REα negative breast tumors and 48 REα positive breast tumors.

Gene	*TNF* mRNA level			
	*REα negative breast tumors*		*REα positive breast tumors*	
***SELE***	0,731 a	**<0,0000001 ^b^**	0,387 a	**0,0065^ b^**
***VCAM***	0,690	**0,0000002**	0,403	**0,0045**
***CSF2/GMCSF***	0,589	**0,00002**	0,373	**0,0089**
***IL8***	0,333	**0,02**	0,202	NS (0,17)
***ICAM***	0,846	**<0,0000001**	0,577	**0,000031**
***CCL2/MCP1***	0,640	**0,000002**	0,578	**0,000029**
***CXCL1/GRO1***	0,494	**0,0004**	0,300	**0,036**
***RELB***	0,858	**<0,0000001**	0,619	**0,000006**
***TNFAIP3/A20***	0,815	**<0,0000001**	0,593	**0,000024**
***IL1A***	0,489	**0,0005**	0,248	NS (0,086)
***IL1B***	0,772	**<0,0000001**	0,477	**0,0007**
***BCL2A1***	0,760	**<0,0000001**	0,434	**0,0022**
***NFKB2***	0,826	**<0,0000001**	0,570	**0,000038**
***NFKB1A***	0,705	**<0,0000001**	0,592	**0,000018**
***CSF1***	0,851	**<0,0000001**	0,427	**0,0026**
***NFKB1***	0,691	**0,0000002**	0,292	**0,041**
***CD40***	0,792	**<0,0000001**	0,433	**0,0022**
***IL6***	0,307	**0,03**	0,275	NS (0.056)
***TNFRSF10B***	0,655	**0,000001**	0,346	**0,015**
***MKI67***	0,079	NS (0,60)	−0,036	NS (0.83)
***ESR1***	-	**-**	−0,063	NS (0.63)

**^a^**Spearman correlation coefficient. **^b^**P value, Spearman rank correlation test.

**NS**, not significant.

**Table 11:** Statistical analysis of mRNA expression of genes in 96 breast tumors relative to relapse.

	*P-value* a	
	*REα negative breast tumors*	*REα positive breast tumors*
**Metastases**		
***Yes***	19	27
***No***	29	21
**Genes**		
***TNF***	0.60 (NS)	0.59 (NS)
***SELE***	0.83 (NS)	0.99 (NS)
***VCAM***	0.92 (NS)	0.78 (NS)
***CSF2/GMCSF***	0.11 (NS)	0.099 (NS)
***IL8***	0.72 (NS)	**0.022**
***ICAM***	0.38 (NS)	0.098 (NS)
***CCL2/MCP1***	0.99 (NS)	0.13 (NS)
***CXCL1/GRO1***	0.90 (NS)	0.33 (NS)
***RELB***	0.89 (NS)	0.45 (NS)
***TNFAIP3/A20***	0.62 (NS)	0.49 (NS)
***IL1A***	0.62 (NS)	0.27 (NS)
***IL1B***	0.51 (NS)	0.55 (NS)
***BCL2A1***	0.25 (NS)	0.78 (NS)
***NFKB2***	**0.039**	0.34 (NS)
***NFKBIA***	0.94 (NS)	0.61 (NS)
***CSF1***	0.43 (NS)	0.70 (NS)
***NFKB1***	0.65 (NS)	0.84 (NS)
***CD40***	0.41 (NS)	0.53 (NS)
***IL6***	0.65 (NS)	0.78 (NS)
***TNFRSF10B***	0.21 (NS)	0.66 (NS)
***MKI67***	0.26 (NS)	0.56 (NS)
***ESR1***	**-**	0.059 (NS)

a Log-rank Test.

### Prognostic value of TNF-α mRNA and of the 19 putative TNF-inducible gene mRNAs in human breast tumors

We estimated the prognostic value of the *TNF-α* mRNA and the 19 other identified putative TNF-α-inducible gene mRNAs in our series of 96 breast tumors (48 ERα-positive and 48 ERα-negative tumors). In the cohort of 48 ERα-negative breast tumors, nineteen patients relapsed, whereas in the cohort of 48 ERα-positive breast tumors, twenty-seven patients relapsed (**Table 11**). We used univariate analysis (log-rank test) to study the prognostic value of these 20 genes. For each gene, the 48 ERα-positive (or –negative) breast tumors were divided into two equal groups of 24 tumors with ‘low’ and ‘high’ mRNA levels. Univariate analysis showed that only low expression level of *NFKB2* in the cohort of 48 ERα-negative breast tumors and high expression level of *IL8* in the cohort of 48 ERα-positive breast tumors correlated with significantly shorter metastases-free survival, but at the limit of the significance (p = 0.039 and p = 0.022, respectively) (**Table 11**).

## Discussion

This study documents, for the first time, the NF-κB gene expression signature in two different subtypes of TNFα-stimulated endothelial cells, especially in HMEC which originate from the microcirculation and have been much less studied as compared to HUVEC. Twenty genes were up-regulated 5-fold or more (until 1200-fold) in TNFα-stimulated endothelial cells, in a concentration- and time-dependent manner. The up-regulated genes include those controlling cytokines, chemokines and adhesion molecules, relevant to inflammation and apoptosis, as well as factors involved in the immune system. The most highly up-regulated genes in TNFα-stimulated endothelial cells are those encoding adhesion molecules and cytokines/chemokines (*i.e.,* TNF-α, IL-8, MCP-1 and GM-CSF/CSF2). Novel observations concern the up-regulation of *TNFAIP3, CXCL1/Gro1, GM-CSF/CSF2* and *MCF/CSF1*, *TNFRSF10B/Trail R2 (DR5), CD40* and *TNF* itself in TNFα-stimulated endothelial cells *via* NF-κB, as well as selective expression of NF-κB components (*RELB*, *NFKB1* and *NFKB2*). Interestingly, while TNFR1 was involved in all the regulations observed, some of the regulated genes were shown to be regulated through both TNFR1 and TNFR2. Moreover, the correlations identified in our *ex vivo* cellular model (endothelial cells) between the expression of TNF-α mRNA and the expression of 19 putative TNF-α- inducible NF-κB target genes were also observed in human breast tumors.

The up-regulation of adhesion molecules (VCAM-1, ICAM-1 and E-selectin) and pro-inflammatory cytokines (IL1α, ILβ and IL-6) in TNFα-stimulated endothelial cells through NF-κB agrees with previous findings reported in HUVEC [16]-[18].IL-6 expression was poorly modulated by NF-κB activation in these cells. Interestingly, our results show that TNF-α strongly up-regulates *TNF-α* itself (but not *TNFR1* and *TNFR2* expression), as well as the expression of *DR5* (*TNFRSF10B* or *TRAIL-R2*) and *CD40*, two additional members of the TNF/TNF receptor superfamily involved in the immune system [3]. Such induction occurs through TNFR1 and TNFR2 and *via* NF-κB activation. Interestingly, DR5 has been detected in endothelial cells derived from blood brain barrier (HBEC), where it released MMP9 upon activation [37].

The chemokines IL-8/CXCL8, CXCL1/Gro1, CCL2/MCP-1, GMCSF/CSF2 and MCF/CSF1 were significantly up-regulated more than 5 fold in HMEC and HUVEC. Their expression was up-regulated as early as 30 min-1 h and sustained for up to 24 h, as previously described for IL-8 and CCL2/MCP-1 in HUVEC [15], [38]. The up-regulation of IL-8 through NF-κB has not been described before in TNF-α-induced endothelial cells. IL-8, CCL2/MCP-1 and CXCL1/Gro1 facilitate the subset specific recruitment of neutrophils, monocytes, T lymphocytes or NK cells. In addition to their chemotactic activity, the three chemokines possess angiogenic activity *in vitro* [39] and are involved in the regulation of tumoral angiogenesis such as in breast cancer [40]-[41]. Our study further shows for the first time that GM-CSF/CSF2 and MCF/CSF1 expression is induced through NF-κB in HMEC. CSF1 has been previously shown to be induced in HUVEC by endotoxin [42]-[43]. This chemokine promotes differentiation of hematopoietic cells to mature granulocytes and macrophages. GM-CSF/CSF2 is involved in the recruitment of macrophages in various inflammatory diseases and was recently found to mobilize bone marrow progenitors [44]. This chemokine is also involved in angiogenesis and is a key target of NF-κB in cancer and metastasis [45].

Bcl2A1 (A1) and TNFAIP3 (also known as A20) are anti-apoptotic proteins expressed in endothelial cells [46]-[48], and have also been associated with angiogenesis [49]. Their expression was shown to be up-regulated by TNF-α in both HUVEC and HMEC, in contrast to other classical NF-κB-inducible anti-apoptotic proteins, such as MCL1S, MCL1L, BIRC2 (c-IAP1), BIRC4 (XIAP) and TRAF2. To our knowledge, our study indicates for the first time the up-regulation of *TNFAIP3/A20* gene expression by TNF-α through NF-κB.

In our study, several approaches were used to demonstrate the involvement of the NF-κB canonical activation pathway in TNF-α-induced gene expression in endothelial cells. Our observations showing that genes encoding the kinases IκKα (CHUK), IκKβ (IKBKB) and NEMO/IκKγ (IKBKG) were not up-regulated by TNF-α in endothelial cells indicate that changes in the NF-κB target gene expression is not dependent upon changes in the expression of these kinases. The decreased expression of the NF-κB-mediated genes in TNF-α treated HMEC in the presence of Bay11–7082 (a specific inhibitor of cytokine-induced IκBα phosphorylation), is in agreement with the involvement of this pathway. It is also in agreement with preliminary experiments showing the time-dependent phosphorylation of IκBα and nuclear translocation of p50 (NF-κB1) in TNF-α endothelial cells (L Michel and M Applanat, *not shown*). The most direct demonstration that TNF-α-induced gene expression in HMEC depends on NF-κB activation arises from RelA knock-down experiments, RelA representing the major NF-κB activity in HMEC stimulated by TNFα [50] (see Fig. 3). Nonetheless, the expression of *TNFRSF10B* is not significantly down-regulated by RelA knock-down as compared to the effect observed with the chemical inhibitors.

The significance of the selective increase of expression of *RELB*, *NFKB1* and *NFKB2/p52*, Rel/NF-κB family members, but not *RelA* and *c-rel,* which is demonstrated for the first time in TNFα-stimulated endothelial cells, is unknown. These up-regulations occur at both the mRNA and protein levels, and correlate with nuclear translocation of p52 *(not shown).* These findings suggest that heterodimers NF-κB2 (p52)/RelA or p52/p52 could also favour TNF-α signalling in endothelial cells. Of note, TNFRSF10B/DR5 has previously been shown to be activated by p52 in some context [51]. The non canonical pathway is known to be activated by stimuli such as lymphotoxin-β, CD40L and BAFF, involves IKKα (IKK1) activation and nuclear translocation of RelB in the form of RelB/p52 and RelB/p50 dimers [9]–[10], [11], [51]–[52].

Our study also contributes to analyze the role of TNFR1 and TNFR2 receptors, which are expressed at a similar level in endothelial cells. The decrease of gene expression is observed in TNFα-stimulated endothelial cells after silencing TNFR1; this receptor is thought to mediate the majority of the biological effects of the TNFα and the classical pathway of TNF-α signalling. However, silencing of TNFR2 also decreases the expression of *IL1B, GM-CSF/CSF2, TNFRSF10B/Trail R2* and *TNF,* suggesting that both TNFR1 and TNFR2 are involved in TNF-α dependent up-regulation of these genes in HMEC.

TNF-α and its related NF-κB transcription factor are described as critical components of tumor progression [20], [22]. NF-κB is deregulated in breast cancer patients and in breast cancer cells [25], [53]–[55]. Several studies have shown that tumor-associated neovascularisation is a prerequisite of rapid growth and metastasis [56]. Although great strides have been made in the elucidation of molecular mechanisms of tumor vasculature, different markers and many essential interactions have to be yet determined. Among genes described in this study, IL8, CCL2/MCP1, CXCL1/Gro1, Bcl2 and TNFAIP3 are known to be pro-angiogenic [38]–[40], [49], [57]. Moreover, one of the consequences for TNF-á activation of tumor endothelial cells is also to recruit numerous cells through chemokines and adhesion molecules, including monocytes, neutrophils, fibroblasts and immune cells inside the tumor.

Our study provides for the first time a significant correlation between TNF-α expression and the expression of putative TNF-α-inducible NF-κB target genes (as identified in microvascular endothelial cells) in human breast tumors (**Table 10** **).** The highest correlation between TNF-α and NF-κB related genes was observed for ERalpha negative-, as compared to ERpositive- breast tumors. TNF-α expression and IL8, IL1A and IL6 expression were only correlated in ER-negative, but not in ER-positive breast tumors. A distinct pattern of activation of NF-κB subunits (c-Rel, RelB, p50/ NF-κB1, p52/ NF-κB2, and Iκ-Bα), as well as the absence (or low level) of nuclear p65/RelA has also been previously described in breast tumors, as compared to normal adjacent tissue [22], [48]. Altogether, these data suggest that dysregulated/TNF-α stimulated endothelial cells through NF-κB are likely to play an important role in breast cancer.

In conclusion, we describe for the first time the profile of cytokines, chemokines, adhesion molecules and NF-κB components induced in endothelial cells stimulated by TNF-α through TNFR and NF-κB. This study provides a NF-κB gene signature associated with TNF-α stimulation in endothelial cells. Some of the newly identified regulated genes, such as those involved in the recruitment of bone marrow or endothelial cell progenitors, will be further explored in a mouse cancer model. The cytokines, chemokines, adhesion molecules and angiogenic factors, as well as factors involved in the immune system, that we have identified in TNF-α stimulated endothelial cells may also promote tumor progression in vivo.
